# Non-infectious rhinitis is more strongly associated with early—rather than late—onset of COPD: data from the European Community Respiratory Health Survey (ECRHS)

**DOI:** 10.1007/s00405-020-05837-8

**Published:** 2020-02-11

**Authors:** Joel Bergqvist, Anders Andersson, Linus Schiöler, Anna-Carin Olin, Nicola Murgia, Mogens Bove, Christer Janson, Michael J. Abramson, Bénédicte Leynaert, Dennis Nowak, Karl A. Franklin, Isabelle PIN, Torgeir Storaas, Vivi Schlünssen, Joachim Heinrich, Johan Hellgren

**Affiliations:** 1 Department of Otorhinolaryngology, Head and Neck Surgery, Institute of Clinical Sciences, Sahlgrenska Academy, University of Gothenburg, Gröna Stråket 9, 413 45 Gothenburg, Sweden; 2 COPD Center, Institute of Medicine, Sahlgrenska University Hospital, University of Gothenburg, Gothenburg, Sweden; 3 Department of Occupational and Environmental Medicine, Sahlgrenska Academy, University of Gothenburg, Gothenburg, Sweden; 4 Section of Occupational Medicine, Respiratory Diseases and Toxicology, University of Perugia, Perugia, Italy; 5 Department of ENT and Oral Maxillofacial Surgery, NU Hospital Group, Trollhättan, Sweden; 6 Department of Medical Sciences: Respiratory, Allergy and Sleep Research, Uppsala University, Uppsala, Sweden; 7 Department of Epidemiology and Preventive Medicine, Monash University, Melbourne, Australia; 8 Inserm UMR1152, Pathophysiology and Epidemiology of Respiratory Diseases, University of Paris, Paris, France; 9 Institute and Clinic for Occupational, Social and Environmental Medicine, University Hospital, LMU Munich, Munich, Germany; 10 Department of Surgery and Perioperative Sciences, Surgery, Umeå University, Umeå, Sweden; 11 Pneumologie Pédiatrique, Antenne Pédiatrique du CIC, Grenoble, France; 12 SKS/RAAO-Helse Vest, Haukeland universitetssjukehus, Bergen, Norway; 13 Department of Public Health, Danish Ramazzini Centre, Aarhus University and the National Research Centre for the Working Environment, Copenhagen, Denmark; 14 Institute and Clinic for Occupational, Social and Environmental Medicine, Comprehensive Pneumology Center (CPC), University Hospital, LMU Munich, Munich, Germany

**Keywords:** Epidemiology, COPD, Rhinitis, Spirometry, Co-morbidity

## Abstract

**Purpose:**

Chronic obstructive pulmonary disease (COPD) is associated with several co-morbidities and non-infectious rhinitis (NIR) has emerged as a new possible co-morbidity. The primary aim of this study is to confirm a previously reported association between NIR and COPD in a multicentre population over time. The secondary aim is to investigate the course over time of such an association through a comparison between early- and late-onset COPD.

**Methods:**

This study is part of the European Community Respiratory Health Survey (ECRHS). A random adult population from 25 centres in Europe and one in Australia was examined with spirometry and answered a respiratory questionnaire in 1998–2002 (ECRHS II) and in 2008–2013 (ECRHS III). Symptoms of non-infectious rhinitis, hay fever and asthma, and smoking habits were reported. Subjects reporting asthma were excluded. COPD was defined as a spirometry ratio of FEV_1_/FVC < 0.7. A total of 5901 subjects were included.

**Results:**

Non-infectious rhinitis was significantly more prevalent in subjects with COPD compared with no COPD (48.9% vs 37.1%, *p* < 0.001) in ECRHS II (mean age 43) but not in ECHRS III (mean age 54). In the multivariable regression model adjusted for COPD, smoking, age, BMI, and gender, non-infectious rhinitis was associated with COPD in both ECRHS II and III.

**Conclusion:**

Non-infectious rhinitis was significantly more common in subjects with COPD at a mean age of 43. Ten years later, the association was weaker. The findings indicate that NIR could be associated with the early onset of COPD.

## Introduction

Chronic obstructive pulmonary disease (COPD) is an inflammatory disease of the lower airways which is seldom symptomatic before the age of 40. COPD is characterised by a chronic limitation of airflow that is not fully reversible after treatment with a bronchodilator. This chronic airflow limitation is due to a combination of small airway disease (obstructive bronchiolitis) and parenchymal destruction (emphysema). Co-morbidities occur frequently in COPD, not only cardiovascular disease [1], but also the metabolic syndrome, osteoporosis, depression, and lung cancer, among others [2]. Recent epidemiological data also show that COPD is associated with non-infectious rhinitis (NIR) [3]. This is further supported by findings of ongoing inflammation in both the upper and lower airways in a limited number of COPD patients [4]. NIR comprises several phenotypes of upper airway inflammation, such as allergic and vasomotor rhinitis, as well as chronic rhinosinusitis. NIR has been associated with poor sleep and impaired rhinitis-specific health-related quality of life assessed with the sino-nasal outcome test 20 (SNOT-20) [5, 6]. Interestingly, COPD patients also have lower scores on the SNOT-20 and thus exhibit an impairment in rhinitis-specific quality of life [7]. Furthermore, treatment with nasal budesonide in patients with COPD has been shown to increase quality of life [8]. COPD and NIR share common risk factors, such as exposure to tobacco smoke, vapours, and fumes [5, 9, 10]. It, thus, appears that NIR has the potential to be a significant co-morbidity in COPD patients.

There is currently a lack of large population-based studies exploring the relationship between COPD and NIR. We found an increase in the 5-year incidence of new-onset NIR (10.8% vs 7.4%, *p* = 0.005) in subjects with COPD compared with controls, in a Swedish random population, assessed with spirometry [3]. One limitation of that study was, however, that it was a single-centre study with a limited follow-up period of 5 years. In the present study, we have included spirometry data from subjects at 26 different centres who were followed for 10 years. The overall aim of the study was to evaluate the relationship between NIR and COPD at different age intervals in the same multicentre cohort over time.

## Methods

The European Community Respiratory Health Survey (ECRHS) was started in 1990 and was called ECRHS I. It is a prospective study of a random adult population from 25 centres in Europe and one in Australia (26 centres in total). The cohort was followed up in 1998–2002 (ECRHS II) and again in 2008–2013 (ECRHS III).

At inclusion in ECRHS I, a random sample of 3000 subjects aged 20–44 at each centre was invited to participate using a short respiratory questionnaire (Stage 1). A random sample of 600 of the responders was then invited to a attend a more comprehensive investigation including spirometry and an extensive respiratory questionnaire (Stage 2). It also included the invitation of 100–150 subjects from each centre reporting respiratory symptoms in the short questionnaire.

**Fig. 1:**
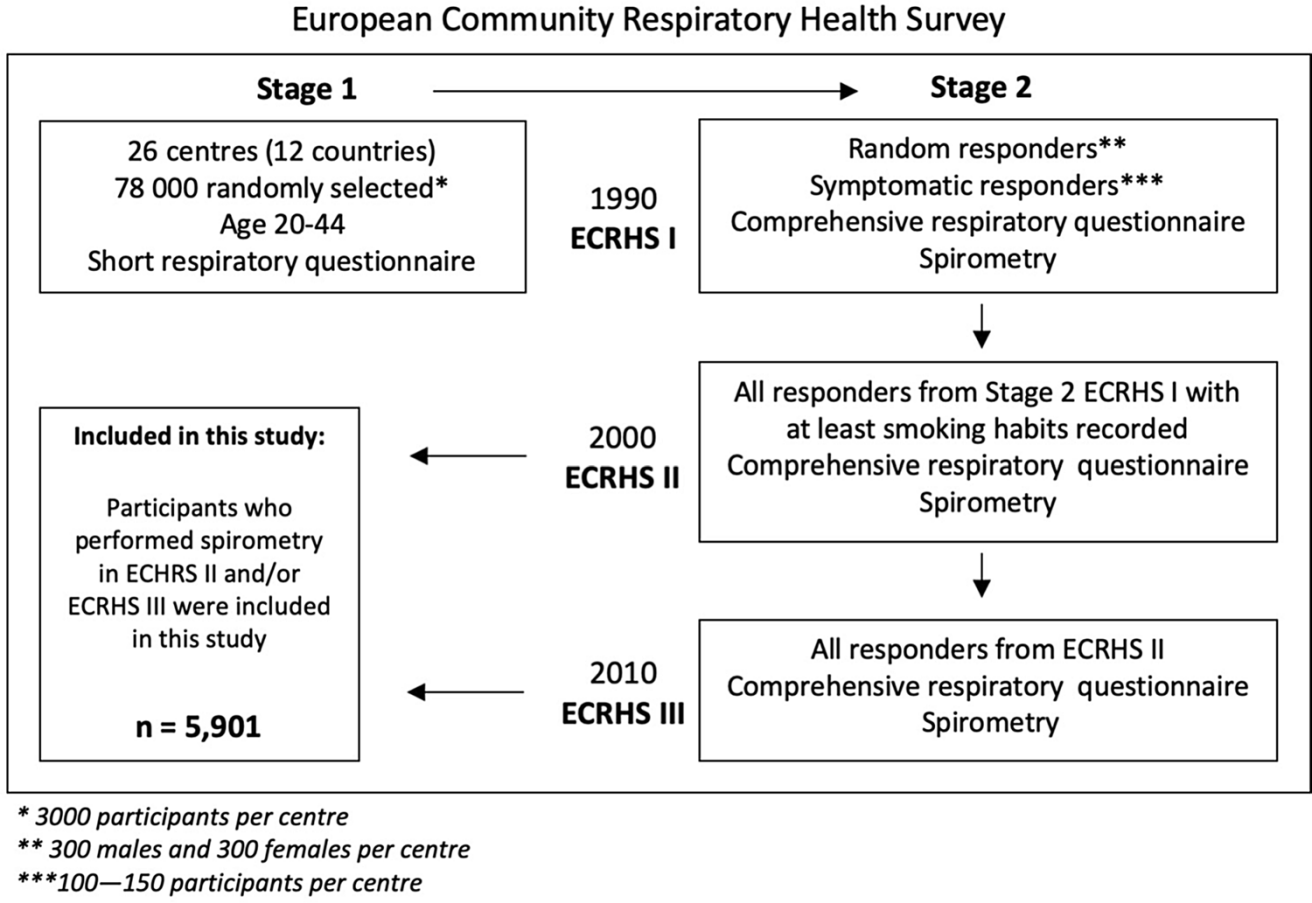
Overview of the ECRHS population

The present study relates to data from 10-year to 20-year follow-ups of ECRHS II and ECRHS III, respectively. All the subjects from ECRHS I (Stage 2) who at least had their smoking status recorded were invited to participate. A flowchart of the ECRHS population is presented in Fig. 1.

In both ECRHS II and III, the subjects answered the same comprehensive respiratory questionnaire and performed spirometry. In ECRHS II, the spirometry was performed without a bronchodilator, according to the study protocol. In ECRHS III, the study protocol was updated and the spirometry was performed before and after a bronchodilator.

Cross-sectional analyses of NIR in relation to COPD were made in both ECRHS II and ECRHS III. Spirometry and questionnaire data were specific to each follow-up in the analyses. In both ECRHS II and III, pre-bronchodilator results were available and were analysed. In ECRHS III, post-bronchodilator results were also available. To minimise the risk of misclassification between asthma and COPD, subjects reporting asthma were excluded (see below). We also analysed new-onset NIR during the 10-year period between ECRHS II and III in relation to having COPD or no COPD at baseline. In a multilevel regression model, we calculated risk factors for having NIR in both ECRHS II and ECRHS III, adjusted for COPD (pre-bronchodilator results in both ECRHS II and III), smoking, age, BMI, and gender.

Chronic obstructive pulmonary disease (COPD) was defined according to the global initiative for chronic obstructive lung disease (GOLD) [2], as an FEV_1_/FVC of < 0.7. Spirometry was conducted according to clinical standards and has been thoroughly described elsewere [11]. Non-infectious rhinitis (NIR) was defined as an affirmative answer to the question: “Have you had a problem with a blocked nose, runny nose, or sneezing when you did not have a cold or the flu during the last 12 months?”. Questions on specific nasal symptoms have previously shown a specificity of 91% and a sensitivity of 96% compared with in-depth interviews on rhinitis [12]. Asthma was defined as an affirmative answer to both “Have you ever had asthma?” and “Have you suffered from asthma attacks within the last 12 months”. Current smoker was defined as a positive answer to the questions: “Have you smoked for as long as a year?” and “Do you smoke now, as of 1 month ago?”*,* while ex-smoker was defined as a positive answer to the first question and a negative answer to the second question. Never-smoker was defined as a negative answer to the question: “Have you ever smoked for as long as a year?”. Hay fever was defined as a positive answer to the question: “Do you suffer from hay fever or other allergies with similar symptoms?”.

### Statistical analyses

Descriptive statistics are presented as the means and standard deviations or as a percentage of total. Chi-square tests were used for univariate analysis. Multilevel logistic regression was used to calculate odds ratios (and 95% confidence intervals) for having NIR, with centre as a random effect to account for within-centre clustering. All analyses were performed using SAS 9.4 (SAS Institute Inc, Cary, NC, USA).

## Results

A total of 84.3% of the eligible subjects in ECRHS II performed spirometry and, in ECRHS III, the corresponding figure was 82.6% (see Table 1).

**Table 1:** Spirometries per centre as a percentage of total eligible subjects included in the study, *n* = 5901

Spirometry/centre	ECRHS II % (*n*)	ECRHS III % (*n*)
Albacete	83.2 (203)	98.0 (239)
Antwerp city	78.3 (119)	90.1 (137)
Antwerp south	87.4 (132)	81.5 (123)
Barcelona	70.9 (151)	66.7 (142)
Basel	84.7 (352)	90.9 (379)
Bergen	96.5 (326)	94.4 (319)
Bordeaux	83.0 (88)	59.3 (63)
Erfurt	99.0 (193)	92.3 (180)
Galdako	84.7 (326)	82.6 (318)
Gothenburg	72.0 (208)	79.9 (231)
Grenoble	82.3 (298)	69.9 (253)
Hamburg	97.3 (178)	89.1 (163)
Huelva	92.3 (144)	93.0 (145)
Ipswich	87.2 (143)	90.2 (148)
Melbourne	86.0 (252)	84.3 (247)
Montpellier	78.7 (96)	66.4 (81)
Norwich	93.3 (140)	88.7 (133)
Oviedo	89.2 (165)	92.4 (171)
Paris	76.9 (256)	64.0 (213)
Pavia	98.7 (76)	89.6 (69)
Reykjavik	95.5 (385)	85.1 (343)
Tartu	36.6 (48)	88.6 (116)
Turin	100 (81)	70.4 (57)
Umea	82.0 (223)	84.6 (230)
Uppsala	76.1 (306)	75.9 (305)
Verona	89.7 (87)	78.4 (76)
Total	84.3 (4976)	82.6 (4881)

Baseline data for the study population are shown in Table 2. The mean age of the subjects was 43 years in ECRHS II and 54 years in ECRHS III. The prevalence of COPD in the population after excluding subjects with asthma was 5.1% before bronchodilation in ECRHS II and 13.8% before and 8.7% after bronchodilation in ECRHS III (Table 2). In ECRHS II, NIR was significantly more common among subjects with COPD compared with subjects with no COPD (48.9% vs 37.1%, *p* = 0.001) (Table 3). In ECRHS III, there was no significant difference in the prevalence of NIR in subjects with or without COPD when excluding subjects with asthma. In addition to FEV1/FVC < 0.7, the lower limit of normal (LNN) was calculated and tested in all analyses, but the result did not change (data not shown).

**Table 2:** Lung function, baseline variables, and smoking habits

Variable	ECRHS II pre-bronchodilator	Mis.	ECRHS III pre-bronchodilator	Mis.	ECRHS III post-bronchodilator	Mis.
*n*	4976	925	4881	1020	4642	1259
Mean age, (years) (SD)	43 (7.1)	×	54.1 (7.1)	×	54 (7.1)	×
Female (%)	52	×	53	×	52	×
BMI, (kg/m^2^) (SD)	25.5 (4.3)	22	27.1 (4.9)	33	27.1 (4.9)	27
FEV_1_/FVC (SD)	0.80 (0.06)	×	0.78 (0.07)	×	0.78 (0.06)	×
Asthma (%)	6.8	6	6.9	14	6.8	12
Hay fever (%)	32.2	16	34.0	21	33.8	20
NIR (%)	41.2	41	42.2	30	42.2	28
Current smoker (%)	26.5	8	17.6	18	17.6	21
Ex-smoker (%)	29.3	8	37.3	18	37.4	21
Never-smoker (%)	44.3	8	45.1	18	45	21
Asthma excluded						
*n*	4624	1277	4516	1385	4302	1599
Mean age, (years)	43 (7.1)	×	54.2 (7.1)	×	54.2 (7.1)	×
FEV_1_/FVC (SD)	0.81 (0.06)	×	0.76 (0.06)	×	0.79 (0.06)	×
COPD (%)	5.1	×	13.8	×	8.7	×
NIR (%)	38.6	39	39.7	22	39.7	21

Study population *n* = 5901 in 1998–2002 (ECRHS II) and/or in 2008–2013 (ECRHS III). Pre-bronchodilator and post-bronchodilator denote spirometry modality in ECRHS II and III, respectively

*ECRHS* European Community Respiratory Health Survey, *Mis.* missing

**Table 3:** The prevalence of non-infectious rhinitis (NIR) in subjects with and without COPD (spirometry: pre-bronchodilator FEV1/FVC < 0.7) in ECRHS II and ECRHS III

	NIR, (%) (*n*)			*p*
	All	COPD	No COPD	
ECRHS II pre-bronchodilator				
No exclusion	41.2 (2033)	53.9 (166)	40.4 (1867)	< 0.001
Asthma excl.	38.6 (1771)	48.9 (114)	37.1 (1657)	0.001
ECRHS III pre-bronchodilator				
No exclusion	42.2 (2049)	46.1 (336)	41.6 (1713)	0.02
Asthma excl.	39.7 (1786)	41.4 (256)	39.5 (1530)	Ns (0.37)
Post-bronchodilator				
No exclusion	42.2 (1946)	44.3 (198)	41.9 (1748)	Ns (0.34)
Asthma excl.	39.7 (1700)	39.4 (146)	39.7 (1554)	Ns (0.88)

**Fig. 2:**
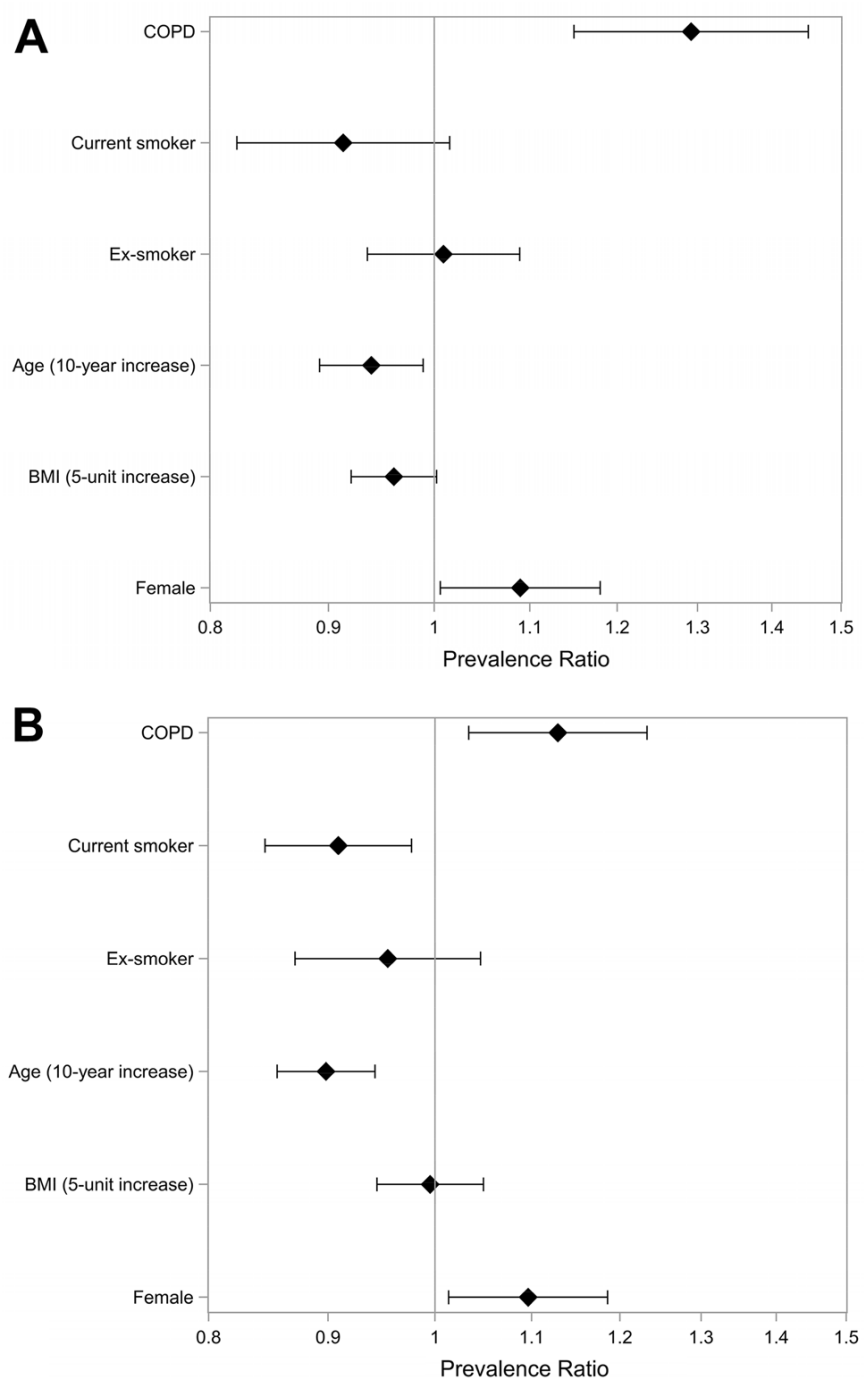
**a** ECRHS II and **b** ECRHS III. Multilevel regression analysis. Prevalence ratio and 95% confidence intervals for NIR in relation to COPD, smoking, age, BMI, and gender. Subjects with asthma excluded. Note that never-smoker was used as a reference category among the smoking categories. *BMI* body mass index, *COPD* chronic obstructive pulmonary disease, *NIR* non-infectious rhinitis

In the multilevel regression model, COPD and female gender were independently associated with NIR in both ECRHS II and III (Fig. 2). Current smoking was associated with a significantly lower risk of having NIR in ECRHS II but not in ECRHS III. In the longitudinal analyses of new-onset NIR during the observation period, COPD in ECRHS II was not associated with an increased risk of new-onset NIR (data not shown).

## Discussion

In this study of a large, random, multicentre population, NIR during the past 12 months was significantly more prevalent in subjects with COPD at a mean age of 43 than in subjects without COPD. Ten years later, the association between NIR during the past 12 months and COPD was still present but weaker. The findings indicate that there is an age-dependent association between NIR and COPD, coinciding with the age interval in which COPD starts appearing in the population.

This study is based on the European Community Respiratory Health Survey, which is a prospective, adult, population cohort followed up at specific time intervals during the past 30 years. One major advantage of this cohort is the multicentre design involving a total of 26 different centres. The multicentre design of this study should have reduced systematic bias which could, otherwise, have been a limitation in a single-centre study of COPD and NIR. A second major advantage is the availability of spirometry data from a large population performed 10 years apart. To our knowledge, this is the largest study exploring the relationship between upper airway inflammation and COPD defined by spirometry data.

The main finding in this study that NIR was significantly more prevalent in subjects with COPD than in subjects without COPD, at a mean age of 43, is both new and interesting. This is an age interval at which COPD starts increasing in prevalence and NIR as a co-morbidity could, therefore, be of importance for the onset of COPD. This is further supported by the multilevel regression model in which NIR remained significantly associated with COPD in ECRHS II, as well as 10 years later, even though the association was weaker. It could indicate a shared age-dependent airway vulnerability in both NIR and COPD which may vary independently over time. Another explanation could be that subjects with COPD and NIR become less aware of their nasal symptoms as their COPD progresses and their NIR, thus, becomes a secondary problem later in life. In our previous study from a single centre, we found that having COPD at baseline was an individual risk factor for developing NIR during a 5-year follow-up [3]. In this present study, we were unable to find the same risk, but the subjects were 5–8 years older at follow-up in this study.

The NIR definition includes both allergic and non-allergic rhinitis. The rationale for using NIR to define upper airway inflammation in this study is that both allergic and non-allergic rhinitis have previously been identified as risk factors for asthma and could, thus, also be associated with COPD. Furthermore, the possible mechanism linking NIR to COPD remains unknown, which justifies the use of a wider definition of rhinitis in this epidemiological study. We found a prevalence of NIR in the population that is comparable to that of others, indicating that our random sample is representative [5]. COPD was defined according to the GOLD criteria as a spirometry result of FEV1/FVC < 0.7. When comparing the results from the present-study population with other population-based data, the prevalence of COPD (6.2–15.0%) was similar to that in other studies [13].

The main analysis was conducted on pre-bronchodilator data in both ECRHS II and III, due to the absence of spirometry data post-bronchodilator in ECRHS II. When comparing ECHRS II and III using the very same method (i.e., COPD definied as “pre-bronchodilator” and “asthma excluded”), we found an association in the first study and a weaker association in the follow-up. The number of COPD subjects was also increased in 10 years which was expected. The present method according to the GOLD guidelines to avoid the misclassification of asthma as COPD is based on post-bronchodilator results. When comparing these two different methods in ECHRS III to estimate the COPD population without asthma, there are a smaller number of COPD subjects when using the post-bronchodilator results, but, importantly, the results stay the same. We, therefore, believe that both the definition and the comparison which we have used are valid in the absence of post-bronchodilator data in ECRHS II.

It is important to point out that our results do not indicate a causal relationship between NIR and COPD. The etiological relationship between upper and lower airway inflammation is still not fully understood. NIR has previously been strongly linked to asthma [14]. Several hypotheses have been suggested. It is logical to believe that a local inflammation caused by exposure to noxious particles and gases such as tobacco smoking affects both the upper and lower airways at the same time and is related to the development of both NIR and COPD [5, 9, 10]. In the multilevel regression model, we, therefore, adjusted for smoking and NIR remained associated with COPD*.* An individual predisposition to the harmful effects of inhaled irritants may also play an important role. Since COPD is a systemic inflammatory disease, it is also probable that there is a systemic component in the development of NIR in COPD subjects, but further studies are needed to address this question.

This study has several weaknesses, of which one is the lack of a spirometry reversibility test in ECRHS II. Furthermore, the subjects were not clinically assessed in relation to their NIR and COPD classification. Questionnaire-based studies are also sensitive to “recall bias” and asking about airway symptoms over a long time period may introduce selection bias.

The findings in the present study still significantly strengthen the possibility of a clinically important association between upper and lower airway inflammation in patients with COPD. A better understanding of the relationship between phenotypes and severity relating to both upper and lower inflammatory airway disease in NIR and COPD is needed. It is important for physicians to acquire an increased awareness of co-morbidities in chronic diseases such as NIR and COPD, whether directly linked to disease development or just associated, to reduce the overall burden of disease for patients. Further clinical studies examining the inflammatory patterns are warranted, with a particular emphasis on the early onset of COPD.

## Conclusion

In this study of a large multicentre adult population, non-infectious rhinitis in the past 12 months was significantly more common in subjects with COPD at a mean age of 43. Ten years later, the association between NIR in the past 12 months and COPD was weaker. The findings indicate that there is an age-dependent relationship between NIR and COPD and that NIR often co-exists at the onset of COPD.
